# Mindfulness and devotion in Tantric Yoga: evidence of a strong trait association

**DOI:** 10.3389/fpsyg.2026.1772396

**Published:** 2026-03-19

**Authors:** Richard W. Maxwell

**Affiliations:** Affiliated Psychological Consultants, PC, Ithaca, NY, United States

**Keywords:** Ananda Marga, Buddhism, devotion, meditation, mindfulness, spiritual, Tantra, yoga

## Abstract

Meditation research has focused primarily on secular forms of mindfulness meditation rather than spiritual forms of meditation, and rarely on devotion in meditation. This study sought to gain broader understanding of the psychological characteristics (mindfulness, mysticism, self-actualization, positive affect and negative affect) and practice characteristics (minutes per day and years of practice) that are associated with devotion within a form of Tantric Yoga meditation. A cross-sectional correlational research design was used to examine questionnaire responses to standardized psychological tests, and information concerning meditation practice. Devotion was found to have the strongest partial correlations (age removed) with trait mindfulness, and trait mindfulness was the only variable to predict devotion in an exploratory multiple regression. This strong relationship between devotion and trait mindfulness may arise from the shared value of developing a one-pointed focus of attention within secular and traditional Buddhist practices, and within the bhakti (devotional) meditation practices of Tantric Yoga.

## Introduction

Early in the expansion of Buddhism into Western societies, especially in secular academic research, mindfulness became primarily defined as a process of concentration and awareness. For example, “Concentration requires one-pointedness, a unified mental focus…. Awareness must be free of thinking, categorizing, judging, accepting, and any other cognitive process and just simply observe the contents and processes of the mind” (Mikulas, 2015, pp. 398–399). This approach facilitated the development and use of secularized versions of mindfulness for therapeutic practices (e.g., Kabat-Zinn, 1990; Segal et al., 2002). As a result, secular tests of mindfulness, including the Cognitive and Affective Mindfulness Scale-Revised (CAMS-R) used here, were developed to evaluate the effectiveness of those mindfulness-based practices. (The decision to use the CAMS-R is explained in the Methods section.) Many secular tests, such as the CAMS-R, measure a set of specific mindfulness-related characteristics present across situations and time (Sala et al., 2020). They can be useful for a degree of understanding, but fall short of being a full expression of the nature of mindfulness (Van Dam et al., 2018). Therefore, representations of mindfulness derived from the CAMS-R are described as “trait mindfulness,” representing a general mindfulness disposition.

Traditional Buddhist perspectives concerning mindfulness are diverse but include ethical considerations. The limited attention given to traditional ethical guidelines within Western approaches to mindfulness led one Buddhist monk to express concern that “the contemplative challenge might be reduced to a matter of gaining skill in certain techniques, dispensing with such qualities as faith, aspiration, devotion, and self-surrender” (Bodhi, 2011, p. 35). Bodhi further expressed that discrimination, evaluation, and judgment must be utilized at times so that (“right”) mindfulness can be coordinated with “right view” and “right effort” as components of the Noble Eightfold Path of Buddhism. Huxter (2015, p. 30) noted that, “Long-term Buddhist meditation practitioners have mixed feelings about the popularization and commodification of mindfulness.” The emergence of the term “McMindfulness” exemplifies this concern (Purser, 2019). While offering a beneficial tool for health, the potential depth and broader value of mindfulness may be overlooked when mindfulness is defined too narrowly and even used for financial gain.

Beyond secular forms of meditation, an analysis of the 2012 National Health Interview Survey (*n* = 34,525) demonstrated that approximately 3.1% of the U. S. population (equivalent to nearly 10 million people) practiced what was called a “spiritual” meditation, defined as “developing a deeper understanding of spiritual/religious meaning and connection with a higher power” (Burke et al., 2017, p. 3). While limited, research on spiritually oriented meditation has demonstrated a variety of diverse benefits, including lower anxiety, stronger positive mood and greater pain tolerance (Wachholtz and Pargament, 2005); improved quality of life, spiritual well-being, spiritual faith, and meaning/peace (Bormann et al., 2006); greater effectiveness in managing migraine pain (Wachholtz et al., 2017); stronger well-being, generativity, and emotional stability (Klein et al., 2016); and better mental health, including a higher level of spiritual experiences (Wang et al., 2023). In contrast to the preponderance of meditation research that has been conducted on novice practitioners, it has been argued that for the nature of advanced meditation to be understood, spiritual perspectives must be included in what is examined (Sparby and Sacchet, 2025).

While Western research has primarily associated mindfulness with Buddhist practices, trait mindfulness measures have been meaningfully used in evaluations of Yogic forms of meditation (Büssing et al., 2012; Gaiswinkler and Unterrainer, 2016; Patel et al., 2018; Maxwell and Katyal, 2022). The relationship between Yoga and mindfulness was sufficiently strong that Jon Kabat-Zinn noted that some of the roots of his original secular mindfulness-based stress reduction (MBSR) were from Yoga traditions, including those of J. Krishnamurti, Ramana Maharshi and Vedanta (Kabat-Zinn, 2011).

Yogic approaches using multiple integrated techniques that are spiritually oriented may have the potential to reveal benefits beyond those present for novice learners of secular meditation. For example, a study assessing a six-month intensive Yoga program, including yoga asanas, meditation, and training in the cultivation of positive qualities, reported significantly elevated spirituality, trait mindfulness (using the Freiburg Mindfulness Inventory, FMI), and mood (Büssing et al., 2012). A more recent study, which was based on a multifaceted Yogic practice, demonstrated that the greatest benefits to individual well-being came from a combination of ethical education and mantra meditation (using a sound, word, or phrase to facilitate a meditative process), followed by a combination of physical yoga, ethical education and mantra meditation. A modest effect was observed for the physical yoga and mantra meditation group, but the mantra meditation alone demonstrated no change in well-being (Matko et al., 2021). Trait mindfulness, also using the FMI, has been found to be correlated with devotional spirituality [using the Daily Spiritual Experience Scale (DSES)] and positive but not negative spiritual experiences (Kohls et al., 2009).

Devotional practices have traditionally been a component of spiritually oriented meditation. In modern literature, devotional practices have been defined as “activities performed for the purpose of focusing one’s attention on the divine” (Wuthnow, 2003, p. 313). Devotion can be found within the history of many religious and spiritual traditions, including Christianity (Hurtado, 2000; Frantzen, 2005; Keating, 2006; Frederick and White, 2015; Papazoglou et al., 2021), Sufism (Khanam, 2011; Werbner, 2017; Saeidi, 2023; Khan, 2024), Hasidic Judaism (Michaelson, 2009; Elior, 2012; Maayan, 2019), and some Buddhist settings (Clarke, 1943; Rinpoche, 1994; Dean, 2019). Buddhist devotion has been ardently expressed in Tibetan Tantric Buddhism. Rinpoche (1994, p. 138) considered devotion “the purest, quickest and simplest way to realize the nature of our mind and all things.” Other more nuanced forms of devotion occur in relation to Theravada Buddhist shrines in Sri Lanka (Clarke, 1943, p. 370), explained as “Gratitude… and devotion to a Master who has left them an example so lofty that its memory cannot perish, but remains as a vivid presence and an enduring value though he has passed forever away.”

The devotional (bhakti) practices within some forms of Yoga have also had a long history (Ánandamúrti, 1992b, 2011a, 2011b; McDaniel, 2018; Oman and Bormann, 2018; Brown, 2021) and are also described strongly. McDaniel (2018) notes that there are many bhakti orientations, the most numerous oriented to “God Vishnu” and his avatars or incarnations (including Krishna and Rama), followed by “God Shiva” and to the goddess Shakti or Devi (often represented by Kali, Durga, and Parvati). Bhakti represents broadly a devotional approach that has run somewhat counter to the more formal aspects of Vedic practices, one that welcomed all, independent of their social status or gender. “Bhakti was first mentioned in the first millennia BC in the Shvetashvatara Upanishad, part of the *Yajur Veda*, the Katha Upanishad and the Bhagavad Gita” (Kiran, 2019, p. 1,748). Kiran goes on to say that the last of *Shvetashvatar Upanishad’*s three epilog chapters, at 6.23, describes that the highest Bhakti (love, devotion to Deva or “God”), should also be directed to the guru (teacher). Somewhat later, approximately 2000 years ago, Patanjali included a component of bhakti in his Yogasūtra (1, 23): “‘Isvara pranidhanat va.’ Or, by devotion to Isvara. …In the yoga system of Patanjali, the concept of Isvara was created due to two points: (1) The relation of the universe of matter, or *Prakriti*, to *Purusha* [Universal Self] (if there be any such relation), (2) as a consequence of (1) an external Guru who teaches yoga to mankind with a view to liberate them, inasmuch as no vidya [force of attraction to the “Universal Self/God/Lord”] can be without its scripture and master!” (Vasantananda, 1966, pp. 26-27). Thus, there is devotional expression not just to the Divine, but also to the Guru, as a vehicle of the Divine.

A Bhakti revolution began in Southern India around the 8th century. By the 15th century, it extended to northern and east India. By the end of 17th century, it had spread throughout India, leading to a profusion of devotional poetry. Since the 17th century, the presence of Bhakti has diminished (Kiran, 2019). This may be related to the arrival of prominent Western influences. Nevertheless, McDaniel considers the majority of Hinduism today to involve some form of *bhakti*, or loving devotion to one or more deities. “This devotion should not merely be ordinary respect and obedience, but *parama prema*, the highest love, which brings a person to perfection. It is passionate longing for God’s presence, and the joy which results from that longing (*premananda*, the bliss of selfless love) brings both immortality and knowledge of the God” (McDaniel, 2018, p. 247). “While the yogic traditions emphasize the importance of religious knowledge, Vaishnavism values love more highly, and the true devotee is one who experiences and expresses that love” (McDaniel, 2018, p. 250). There is a strong influence of bhakti within the Ananda Marga discipline of Tantric Yoga (AMTY) of the participant sample, which among other things, includes an emphasis on singing devotional kirtan, a classic Vaishnava practice.

It has been said (Ánandamúrti, 2024, p. 11), “That which makes mind soft and so strong and strenuous as it may keep itself in a balanced state even in the condition of pain, and creates perpetually a pleasant feeling within, is called love. Devotion is identical with love. They are invariably related with each other. The moment devotion is aroused, love for God comes.” Elaborated further, the conception of the peak form of bhakti, kevalā bhakti (analogous to the parama prema described by McDaniel), has been described as,

“Bhaktir Bhagavato sevā bhaktih prema svarūpinii;

Bhaktirānanda rupā ca bhakti bhaktasya jiivanam.

[Bhakti is service to God; bhakti is the form taken by divine love;

bhakti is the embodiment of bliss; bhakti is the life of the devotee.]

… They remove their individualities, and accept Parama Puruśa as their nearest and dearest one. This is what is called exclusive love for God. This is called kevalā bhakti. And only in this stage does devotion reach its fruition or culmination” (Ánandamúrti, 1994e, p. 209).

AMTY has been described as a Tantric Yoga discipline. The conceptual framework associated with that is elaborated in the beginning of the Methods section. The current study, utilizing participants observing AMTY practices, expands upon recent work (Maxwell and Katyal, 2022), which demonstrated that levels of trait mindfulness were significantly related to greater quantities of daily meditation, in addition to indications of stronger well-being for practitioners within AMTY. Despite the broad presence of devotion in various forms of spiritual meditation, as suggested above, there has been little analytic research on the nature of devotion within meditation. Therefore, the present study examines levels of devotion in relation to a broader set of variables, including trait mindfulness, self-actualization, mystical experiences, positive and negative affect, and time spent meditating. On the basis of the results of other studies of spiritual meditation (Wachholtz and Pargament, 2005; Greeson et al., 2011, 2015; Ivtzan et al., 2013; Maxwell and Katyal, 2022), devotion is expected to be significantly related to greater trait mindfulness, greater self-actualization, greater mystical experiences, increased positive trait affect/decreased negative trait affect, and greater amounts of daily meditation.

## Methods

A cross-sectional correlational research design (Judd and Sadler, 2003; Lau and Kuziemsky, 2016) was used to examine relevant psychological characteristics that might demonstrate features related to devotion.

### Tantric Yoga

It is important to clarify the nature of the Tantric Yoga orientation of AMTY in order to understand the philosophical framework the participants in this study have been provided which may potentially influence their responses on the test items they have given.

First, the Tantric nature of AMTY will be examined. While AMTY did not originate through a particular historical lineage (Vijayananda Avt and Acyutananda Avt, 1993, pp. xiii–xiv), its primary model of seven cakras (although in some circumstances nine are used) and kuṇḍalinii most closely resembles the system prevalent in Shākta Tantra (both Kāliikula and Shriividyā) and within Haṭhayoga, which include a system of seven cakras with the names mūlādhāra, etc. There is little evidence in Tantric literature before the second millennium for this system. It became common in various branches of Shākta Tantra and Haṭhayoga around the year 1,200 CE and can be seen in the *Shāradātilaka* (c. 12th century), *Matsyendrasaḿhitā* (c. 13th-century), *Yoginiihrdaya-diipikā* (14th-century), *Rudrayāmala Uttaratantra* (14th or 15th century). It is a major departure from what is known of earlier Shaeva and Buddhist Tantra. This model became more widely known after the translation of the *Śaṭcakranirūpaṇa* chapter of the *Tattvacintāmaṇi* (16-century) by ‘Arthur Avalon’ in 1913 (Hatley, 2022). This orientation prevailed in Bengal during the time Prabhat Rajan Sarkar (aka, Shrii Shrii Ánandamúrti, his spiritual name), founded and developed Ananda Marga. He states, “The fundamental goal of this sādhanā is to awaken the dormant *jiivashakti* [unit force], known as *kulakuṇḍalinii*, and, after elevating it stage by stage [i.e., chakra by chakra], to merge it in *Brahmabhāva* [Cosmic Consciousness] (Ánandamúrti, 1993b, p. 162).”

“The origins of Tantra are usually said to be around 500 CE (though some scholars point to older precursors). Over the next thousand years, it elaborated theories and practices, which have influenced both philosophical and devotional traditions in Hinduism. The Tantras usually emphasize the gods Shiva (especially in the Kashmiri Kaula tradition), or Krishna (in the Vaishnava Sahajiya tradition), or the goddess Shakti (in the South Indian Shri Vidya and Bengali Shakta tantric traditions). Tantric ideas and rituals have also been influential in Buddhism, especially Tibetan Vajrayana” (McDaniel, 2018, p. 244). By the 10th and 11th centuries, there had been a phasing out of the more ‘transgressive’ Tantric rituals, being replaced by a quest for individual liberation or self-realization (Samuel, 2008). In AMTY, Shiva (e.g., Ánandamúrti, 1982) and Krishna (e.g., Ánandamúrti, 1981) have both been highly respected, although Shakta perspectives have primarily been utilized for conceptions of chakras and kuṇḍalini function (Hatley, 2022). “Patanjali does not mention kundalini, but speaks of the energy of nature flowing abundantly in a yogi (YS IV.2)” (Iyengar, 2002, p. 184) which may be a somewhat analogous older terminology. Iyengar (1979, pp. 439–440) has also noted that in Tantric texts, the kundalinii is a “latent energy” that can be made to “rise from the base of the spinal column piercing the chakras” to “unite with the Supreme Soul”.

McDaniel (2018) considers the medieval *Kulārnava Tantra* to have been an important text for both Kashmiri and Bengali tantric traditions. This is an accurate reflection of AMTY which originated in Bengal. For example, a quote from the *Kulārṇava Tantra* has been used in AMTY literature on meditation (sādhanā) to accentuate the best practice (Ánandamúrti, 1993b, p. 164):

“Uttamo Brahmasadbhāvo Madhyamā dhyāna dhāraṇā;

Japastuti syādhadhamā Mūrtipūjā dhamādhamā.

[Ideation on *Brahma* is the best, *dhyāna* and *dhāraṇā* are second best, repetitious incantation and eulogistic prayer are the worst, and idol worship is the worst of the worst.]” The nature of the “best” is further demonstrated in a translation of an excerpt from the *Mundaka Upanishad 2.2.4*, from the *Atharva Veda*:

“Praṇavo dhanuh sharohyātmā Brahma tallakṣyamucyate;

Apramattena veddhavyaṁ sharavattanmayo bhavet.

A sādhaka who utilizes his or her spiritual practice [sādhanā]as the bow, her or his self [individual subjectivity, Jiivatman] as the arrow, and Parama Puruśa [the Supreme Subjectivity, or Paramātman] as the target, and then tries to pierce the target with undivided attention, attains the supreme goal” (Ánandamúrti, 1994a, pp. 235–236).

Focusing on conceptions of Yoga, Patanjali’s relatively well-known definition of Yoga in *Yogasūtra* (YS), sūtra 1.2 which reads in Sanskrit: “Yogaḥ citta vṛtti nirodhaḥ” is translated, for example, by Vasantananda (1966, p. 3) as: “Yoga is the restraint of mental modifications”. In Patanjali’s version of vrttis, five forms of mental modifications are described (YS 1.6): “correct knowledge, illusion, delusion, sleep and memory” (Iyengar, 2002, p. 56). This term “vrtti” used in YS 1.2 is not the technical jargon of only one specific philosophical or yogic system, and can be found in the *Yogasudhākara* of Sadāshivasarasvatii, the *Parātrishṁikā-vivaraña* of Abhinavagupta, the *Sarva-vedānta-sāra-saṁgraha* and the *Jiivanmuktiviveka* (Hatley, 2022). Ánandamúrti’s (1992b, p. 101) Tantric perspective, differs from Patanjali’s definition of Yoga, “What is Yoga according to Tantra? Tantra defines Yoga as ‘Saṃyogo yoga ity ukto jīvātmaparamātmanoḥ’ [from the *Yogayājñavalkya, 1.15*]. The unification of Jiivātman [individual subjectivity] with Paramātman [Supreme Subjectivity] is termed Yoga”.

While Ánandamúrti (1988a, p. 671) accepts Patanjali’s vrttis as “five broad categories” of vrttis, he emphasizes a different, more detailed conception of vrttis in which each petal on the symbolic chakra lotus flowers represents a vrtti (Sarkar, 1994). The earliest clear precedent for Ánandamúrti’s vrtti conception is a musicological text, the *Saṁgiita-ratnākara* of Shārṋgadeva (mid-13th-century), although Shārṋgadeva did not use the term vrtti. Each petal is said to have a “fruit,” or an associated mental–emotional state (Kitada, 2012). It is likely that Shārṋgadeva drew on older, lost sources (Hatley, 2022). Avalon (1974, p. 138) (aka Sir John Woodroffe) referenced a related text also by Shārṋgadeva, currently lost, the *Adhyātmaviveka*. He gives that as the primary source for the vrtti states he associates with each chakra petal in relation to his commentary and translation of the *Sat-Cakra-Nirupana*. The *Sat-Cakra-Nirupana* was composed a little later than Shārṋgadeva’s work, by “Tantric Purnananda-Svami in year 1577 CE” (Avalon, 1974, p. xiv). Avalon and associates published translations and studies of the Shākta Tantras of Bengal. Shyam Sundar Goswami, also from Bengal, used this same system of chakra petal vrttis, with near identical descriptions of each vrtti to those of Avalon (Goswami, 1999). By the 1920s, there was a large and eclectic body of literature on chakras and yoga in various languages claiming connections between the yogic subtle body and formal anatomy and physiology (Hatley, 2024). That is the environment in which Ánandamúrti developed his synthesis of Tantric Yoga perspectives, similar to Avalon and Goswami, but with significant innovations.

In his chakra petal vrtti system, Ánandamúrti proposed a more specific physiological function, that the activity of the vrttis were related to glandular hormonal secretions that in turn would influence the functioning of the mind. “If there is any defect in the secretion of hormones or any defect in a gland, certain *vrttis* become excited. …If people want to control the excitement of these propensities [to diminish mental fluctuations], they must rectify the defects of their glands” (Ánandamúrti, 2000, p. 23). One way this rectification could be accomplished is through performing appropriate āsanas that had the potential to modify glandular function through the “pressurizing or depressurizing” of local regions in which those glands are situated (Sarkar, 1994, p. 123).

Patanjali’s approach for āsanas is: “Sthira sukham āsanam [YS 2.46] …Āsana is a steady, comfortable posture” (Satchidananda, 2012). Iyengar (1979, p. 40), from a totally different region of India, popularized Hatha Yoga practices and is especially well-known for his detailed teachings concerning āsanas. While agreeing with Patanjali’s definition, his more detailed perspective considers the movements of āsanas to “exercise every muscle, nerve and gland in the body”. By “performing the various āsanas accurately and precisely, each particle of the body is nourished with fresh blood and energy. …All of these reactions from āsanas help soak the body in freshly oxygenated blood” (Iyengar, 2012, p. 154). He also considers āsanas to produce “mental equilibrium” and prevent “fickleness of mind” (Iyengar, 1979, p. 40). Ánandamúrti (1994b, pp. 114–115) provides a similar general description of the benefits of āsanas, as “calm, quiet and easy postures which are held with proper inhalation and exhalation. They exercise the nerves, tissues, glands and organs of the human body. While practicing āsanas one enjoys physical comfort and mental composure”. Research has confirmed that Iyengar’s approach to āsanas has physical health benefits (Williams et al., 2003) and also provides benefits to mental functioning and feeling states (Harner et al., 2010). Despite Iyengar’s interest in the physiology and psychology of yoga āsana practice, he does not appear to describe āsana effects in the manner of Ánandamúrti’s apparently unique expanded role of glandular secretions and more detailed set of vrttis (Sarkar, 1994).

### Participants

Individuals actively practicing meditation taught by AMTY were the participants in this study. Note that since Ánandamúrti’s death in 1990, the organization that he had created has divided into several different groups, each with its own name. The participants in this study were recruited independent of their group affiliation. Therefore, the alternative name AMTY (Ananda Marga discipline of Tantra Yoga) has been used to refer to the common training and practices of these participants.

A total of 72 individuals were recruited. An initial 28 individuals were recruited at three AMTY meditation retreats (one in Albany, NY, October 2008, and two near Asheville, NC, October 2008, and March 2009), at which paper-and-pencil questionnaires beginning with a consent form were administered (Group 1). One person declined to submit the consent form, preferring to remain anonymous, but otherwise submitted a complete questionnaire. Lacking consent, their data were excluded from analysis. One individual failed to supply necessary meditation information and was excluded. At a later point (from 12/23/2015 to 4/11/2017), additional individuals observing AMTY practices were recruited, primarily through personal solicitations and an internal AMTY online forum. Forty-four participants (Group 2) successfully completed a Google Form version of the Group 1 questionnaire. Two individuals completed the Google Form questionnaire twice. Their second submissions were excluded. One individual was judged to have inadequate English skills and was excluded. After those combined exclusions, the data set contained 67 individuals.

After imputation to manage missing data (explained below in the Data Analysis section) and analysis of basic data characteristics, three additional individuals were excluded from Group 1 because of extreme scores. These were identified through an examination of skew and kurtosis among the variables and from extreme values present within box plots. No extreme values were found among Group 2 members. With the removal of those participants, all variables had a skew that was less than ±1.5 and a kurtosis less than ±2.5, meeting recommended guidelines (West et al., 1995). As a result, 64 individuals were included as participants in this study.

All the participants volunteered and completed the questionnaires at their own pace during their personal time. The participants were allowed to skip items or opt out of the study at any point if desired. The consent provided by all participants followed guidelines recommended by the American Psychological Association Ethics Code, section 8.02 (American Psychological Association, 2021). The present research was not conducted within an institutional or medical setting.

The information that was gathered was detailed, extensive, and contrary to the typical culture of this AMTY path, which generally discouraged talking about personal meditation experiences to avoid potential ego inflation. Access was gained to this detailed information solely because the researcher was also a participant within this path. Without the respect and trust that “inside status” generated, the information would not have been shared. This poses potential conflicts in the impartiality of the research process, but no direct comparisons were made with other systems of meditation. Therefore, it is unlikely that this poses a major limitation. This situation is not unique. Poloma (2003) has commented extensively on the access she received due to her similar relationship with the charismatic revival of Pentecostalism. The various ecstatic mystical experiences occurring within Pentecostal services resemble those of the kundalini experiences described in Yoga literature (compare Poloma and Pendleton, 1989; and Poloma, 1998; with Edwards and Woollacott, 2022).

A copy of the questions (Survey-2 Questions) that were used in this study can be found at: https://osf.io/24srp/. Those questions were part of a larger questionnaire designed to be inclusive of prayer practices in addition to meditation. The questionnaire was titled “A Survey of Experiences Arising from Prayer and Meditation.” However, insufficient individuals with prayer or meditation practices outside of AMTY were able to be recruited. Therefore, only individuals actively observing AMTY practices were included in this study. Additionally, various personal experiences during meditation and prayer were not included in this study and were analyzed separately.

### Contemplative practices

All of the participants in this study were trained in the style of meditation taught by the Ācāryas (individuals officially trained to teach meditation practices and mantras) within AMTY. While the participants’ training included the first three limbs (Yama and Niyama-ethical practices and āsanas-yoga postures) of Patanjali’s Aṣṭāṅga framework (Larson, 2012; Gard et al., 2014; Katyal, 2022), the time spent associated with those practices was not considered “meditation.” Meditation was considered to comprise the remaining 5 limbs, and included the use of mantras. (The use of mantras for meditation is an innovation in yoga practices and beyond that found in Patanjali’s system.) These higher limbs include prāṇāyāma (breath control), pratyāhāra (withdrawal from externally oriented mental activity), dhāraṇā (concentration or to hold in mind), dhyāna (devotional meditation or to direct the mind to the divine) and samādhi (absorption). This broad interpretation of meditation was taken because the participants’ AMTY meditation practices included combinations of those five limbs which were organized in a manner unique to AMTY and not in a simple step-wise fashion through the eight limbs. For example, in the ideal situation, feelings of devotion would be present within each meditation component, although most central to dhyāna.

All the participants were solely observing AMTY meditation practices at the time of the data collection. Assessing the degree of meditation experience was complicated by the presence of some individuals who had practiced other forms of meditation or prayer before adopting the AMTY practices. An inclusive approach was used, believing that prior experience would contribute meaningfully to experiences with AMTY practices. Therefore, to quantify the total number of years of meditation practiced by participants, participants were asked to report up to four different forms of meditation or prayer practices that they had practiced and the number of years spent doing those practices. The total years of all forms of meditation or prayer were summed and called the variable “TtlYear.” The responses included 16 individuals (25%) who had some earlier other experience. Three (5%) had greater time engaged in earlier practices. To capture the current intensity of the practices, the participants were asked to specify the current daily quantity of time (in minutes) typically spent meditating (called the variable “DailyMin”).

### Psychological measures

#### Devotion

The Daily Spiritual Experience Scale (DSES; Underwood and Teresi, 2002) is a 16-item scale. In three different samples, the internal consistency of the DSES, which is based on Cronbach’s alpha (Cronbach, 1951), ranged from 0.91 to 0.95 (Underwood, 2011). The current study had an *α* = 0.92. Half of the DSES items used the word “God.” Administration instructions included the following guidance: “If there is a better word or set of words than ‘God’ that calls to mind the divine or holy for you, then please substitute that when the word ‘God’ is used.” All items were positively worded because negative wording was considered to take on additional meaning beyond the negation. The first 15 items included 6 response categories: never or almost never, once in a while, some days, most days, every day, and many times a day. The following are two examples of items. “I desire to be closer to God or in union with the divine.” “I feel thankful for my blessings.” The 16th item had 4 possible answers (not at all, somewhat close, very close, and as close as possible) to the question “How close do you feel to God?” The original norms for the development of the DSES (Underwood and Teresi, 2002) used a scoring system that is not currently recommended by the first author (Underwood, 2011). Subsequent modifications (Underwood, 2006) recommended to “spread” the 4-point 16th item over a “6-point spectrum” and combine all 16 items into a total score. To achieve this, it was recommended that “not at all” be ranked 1, “somewhat close” 3, “very close” 5, and “as close as possible” 6. After all the items were summed, a mean score was generated by dividing by 16.

The DSES has been generally recognized as a measure of “spirituality,” identifying the frequency of relatively common spiritual experiences (Underwood, 2011). While not overtly utilizing the term “devotion,” the experiences analyzed by the DSES items closely resemble the devotional qualities within AMTY meditation practices. Those qualities are associated with the recognition of the presence of the divine within every aspect of inner and externally oriented experience (Ánandamúrti, 1994d). Therefore, in the current work, the DSES has been defined as a measure of devotion. This innovation is relevant for the current participant sample, but may not be relevant in other circumstances.

#### Mindfulness

The Cognitive and Affective Mindfulness Scale-Revised (CAMS-R; Feldman et al., 2007) measures a set of cognitive characteristics representing fundamental qualities of mindfulness. These included “(1) the ability to regulate attention, (2) an orientation to present or immediate experience, (3) awareness of experience, and (4) an attitude of acceptance or nonjudgment toward experience” (CAMS-R; Feldman et al., 2007, p. 178). These characteristics were derived from a definition of mindfulness produced by a consensus process among a group of academic researchers (Bishop et al., 2004). Since these characteristics did not represent completely the full potential meaning of mindfulness, and since these measured a sustained general level of specific mindfulness-related qualities, the CAMS-R has been referred to as trait measure, or representing trait mindfulness.

The decision to use the CAMS-R was based on its use in an earlier study (Maxwell and Katyal, 2022) arising from multiple considerations. First, the CAMS-R includes more than just the attentional and awareness aspects of mindfulness, such as in the Mindfulness Attention Awareness Scale (MAAS; Brown and Ryan, 2003), adding attitudinal components of acceptance and non-judgment. Second, the Kentucky Inventory of Mindfulness Skills (KIMS; Baer et al., 2004) and the Five Facet Mindfulness Questionnaire (FFMQ; Baer et al., 2008) are substantially longer, both having 39 items. The CAMS-R can be administered more efficiently, having only 12 items, which is beneficial when used as a component in a long questionnaire. Another mindfulness test available at that time, the Toronto Mindfulness Scale (TMS; Lau et al., 2006) is a measure of state mindfulness, seeking a measure of the immediate experience within the past 15 min, reflecting a short-term state of mindfulness, and not trait mindfulness. Last, the Freiburg Mindfulness Inventory [FMI; (Buchheld et al., 2001)] was also available at that time but had not been identified. Thus, the CAMS-R was considered the best choice.

The internal consistency of the CAMS-R is reported to be *α* = 0.76 (Feldman et al., 2007). In the current study, α = 0.79. The CAMS-R is based on the rankings of 12 statements. These are divided into four facets (attention, present focus, awareness, and acceptance), which produce a total score for trait mindfulness when summed. The items are rated on a 4-point scale: rarely/not at all, sometimes, often, and almost always. Examples of CAMS-R questions include “I am easily distracted.” “I am preoccupied by the past.” “I try to notice my thoughts without judging them.” “I can tolerate emotional pain.” Three items are negatively worded, and their scores are inverted.

#### Mystical experiences

The Mysticism Scale (MS; Hood, 1975) is a 32-item instrument intended to assess the quantity/intensity of uncommon spiritual experiences, reflecting the broader concept of mysticism (Klein et al., 2016). The MS has an *α* = 0.91 for a large US sample (Hood et al., 2001). The internal consistency in the current study is α = 0.92. MS was found to involve three factors: extrovertive mysticism, religious interpretation and introvertive mysticism (Hood et al., 1993). The following are examples of each respective factor: “I have had an experience in which all things seemed to be conscious.” “I have had an experience which I knew to be sacred.” “I have had an experience which cannot be expressed in words.” Each item is rated on a scale of 5: definitely true, probably true, cannot decide, probably not true, and definitely not true.” Half of the items are negatively worded, and their scores are inverted. A negatively worded item is “I have never experienced anything to be divine.” Only the MS total score is used in this study. The MS has been found to be significantly correlated with the DSES (Wachholtz and Pargament, 2005).

#### Self-actualization

The Short Index of Self-Actualization (SISA; Jones and Crandall, 1986) is a 15-item scale designed to be an efficient means to measure the concept of self-actualization (Jones and Crandall, 1986). The concept of self-actualization was popularized by Maslow (1943) within his hierarchy of needs, reflecting the transformational development of psychological health (Bland and DeRobertis, 2020). The SISA is highly correlated with an accepted measure of self-actualization, the Personal Orientation Inventory. The SISA has an α = 0.65. Similarly, the present study has an α = 0.64. There are 4 possible responses for each item: agree, somewhat agree, somewhat disagree, and disagree. Eight items are negatively worded, and their scores were inverted. An example of a positively worded item is “I can like people without having to approve of them.” A negatively worded item is “I am bothered by fears of being inadequate.” The SISA has been associated with spiritual qualities (Ivtzan et al., 2013).

#### Affect

The Positive and Negative Affect Schedule (PANAS; Watson et al., 1988) is a measure of affect, including a positive (PosAff) and negative (NegAff) scale. There are various time frame options that may be used for administration. To identify trait affect, a broad timeframe is used: “Indicate to what extent you generally feel this way, that is, how you feel on average.” Internal consistency for this scale timeframe is reported to be α = 0.88 for PosAff and α = 0.87 for NegAff. The data in this study demonstrates α = 0.82 for PosAff and α = 0.92 for NegAff. Each scale has 10 adjectives that are ranked on a 5-point scale: very slightly, a little, moderately, quite a bit, extremely. Examples of PosAff include “alert” and “inspired.” NegAff includes “upset” and “afraid.” Recent research demonstrates that PosAff and NegAff are significantly correlated with AMTY meditation (Maxwell and Katyal, 2022).

### Adjustments

In some instances, participants provided two answers on a single Likert scale item, i.e., two neighboring levels were indicated when only one was requested. Presumably, they could not decide between the two, or both in some way applied for them. In such instances, that ambiguity was resolved by using whichever score was closest to the mean of the levels for that item. In instances in which there were missing data, the missing values were imputed. The details of the imputation process are presented below in the Data Analysis section.

### Data analysis

Statistical analysis of the data was conducted using R (v.4.2.2) statistical software and RStudio (2022.12.0 + 353). For descriptive statistics, partial correlations, and multiple linear regressions, the “psych” (Procedures for Psychological, Psychometric and Personality Research) package was used. The “ltm” (latent trait models) package was used for the measurement of Cronbach’s alpha, and the “BSDA” (basic statistics and data analysis) package was used for t tests. Collinearity was determined by the “VIF” (variable inflation factor) available in the “CAR” (Companion to Applied Regression) package. The variables used for statistical analysis in R (Survey-2 Data.csv) and the data file used for the imputation (Survey-2 for Impute.csv) may be found at: https://osf.io/24srp/.

The “MICE” (multivariate imputation and chained equations) package was used for imputation. A total of 1.48% of the data from the psychological measures used for multivariate statistical analysis were missing. None of the demographic (age, sex and education) or meditation variables (DailyMin and TtlYear) that were included in the imputation process had any missing values. Data imputation was run to facilitate a statistical comparison of the variables. Greater accuracy and less bias in data have been demonstrated for multiply imputed data than for data using the common approaches of pairwise or listwise deletions (Newman, 2014).

The significance levels were corrected when partial correlations and standard two-sample t tests were conducted. The corrections were performed using the “BH” (Benjamini–Hochberg) procedure (Benjamini and Hochberg, 1995), a.k.a. “FDR” (false discovery rate). When partial correlations were used, the effect of age was removed. Age has been shown to influence affect and happiness (Yang, 2008; Carstensen et al., 2011).

## Results

### Preliminary analyses

In this study, there were 21 women (33%) and 43 men (67%) (see Table 1). Group 1 included 5 (22%) women and 18 (78%) men, and Group 2 included 16 (39%) women and 25 (61%) men. The mean age of the participants was 50.6 years (SD 15.6, range 19–75). Group 1 had a mean age of 40.7 years (SD 14.3, range 19–62). Group 2 had a mean age of 56.1 years (SD 13.6, range 22–75). In terms of education, 11 (17.2%) participants had a high school education, 5 (7.8%) had associate’s degrees, 13 (20.3%) had bachelor’s degrees, 24 (37.5%) had master’s degrees, and 11 (17.2%) had doctoral or law degrees.

**Table 1:** Participant sex, age and group.

Variable	Both groups	Group 1	Group 2
Total *N*	64	23	41
Women (%)	21 (33%)	5 (22%)	16 (39%)
Men (%)	43 (67%)	18 (78%)	25 (61%)
Total mean age (SD)	50.6 (15.6)	40.7 (14.3)	56.1 (13.6)
Mean age of women (SD)	49.9 (14.8)	38.2 (14.7)	53.6 (13.3)
Mean age of men (SD)	50.9 (16.2)	41.4 (14.6)	57.7 (14.4)

Sex and age details for participants are presented for the combined groups and the two questionnaire administration approaches (Group 1 with paper and pencil, Group 2 online using Google Forms).

The two forms of data administration (Group 1 and Group 2) were compared to determine if they generated any significant differences among the study measures. Standard two-sample t tests were used to compare these two groups for each variable, first checking for homogeneity of variance. When there was a significant difference between the two variances (twice in 17 comparisons), a Welch t test was used. Significant t test differences were observed for age (*t* = −4.26, *p* < 0.0001) and TtlYear (*t* = −2.7464, *p* < 0.01). Both were expected given the 6–7 year separation in the two administrations and the age difference between the two groups. There was also a lower likelihood of newer (often younger) practitioners being members of the online forum used to solicit participants for Group 2. The significant difference in age between the two groups remained significant, even when significance was adjusted for multiple comparisons using the BH procedure (Benjamini and Hochberg, 1995). With a similar adjustment for multiple comparisons, TtlYear no longer had a significant difference between the two groups. Given the significant difference in age, in addition to other potential influences of age on psychological test variables, all the statistical analyses of the psychological tests took age into account. With that statistical compensation, it was considered reasonable to combine the two groups and analyze the data without considering the style of questionnaire administration.

Given the presence of slightly more than double the number of men compared with women, each variable was also analyzed using two-sample t tests to determine whether there were any significant differences due to sex. This analysis followed the same structure as the one for the two groups. Three variables among the 16 comparisons lacked homogeneity of variance and required the use of a Welch t test. There was no significant difference in sex for any of the variables. Therefore, sex was not included as a separate variable.

### Correlational analysis

To examine basic relationships within the data, a Pearson partial correlation was conducted, removing the age effect. See Table 2. The probabilities were adjusted for multiple measurements. Five significant partial correlations were present for devotion, and the strongest partial correlation was with trait mindfulness (*r* = 0.60, *p* < 0.001). Additional significant partial correlations existed for devotion with self-actualization (*r* = 0.46, *p* < 0.001), positive trait affect (*r* = 0.41, *p* < 0.01), negative trait affect (*r* = −0.34, *p* < 0.05), and DailyMin (*r* = 0.33, *p* < 0.05). Both trait mindfulness and self-actualization also had five significant partial correlations, and the number of minutes of daily meditation (DailyMin) had four. Negative trait affect had four significant partial correlations, and positive trait affect had three. Mysticism had only one, with self-actualization. The number of years of meditation experience (TtlYear) lacked any significant partial correlations.

**Table 2:** A Pearson partial correlation matrix of meditation and psychological measures.

Variable	DailyMin	TtlYear	Mful	PosAff	NegAff	Myst	SA
TtlYear	0.201						
Mful	0.301^*^	0.073					
PosAff	0.196	0.105	0.546^***^				
NegAff	−0.318^*^	−0.009	−0.405^**^	−0.227			
Myst	0.103	0.012	0.271	0.136	−0.008		
SA	0.298^*^	0.109	0.501^***^	0.546^***^	−0.522^***^	0.309^*^	
Dvtn	0.326^*^	0.071	0.603^***^	0.414^**^	−0.342^*^	0.232	0.458^***^

The effect of age was removed, and the probabilities were adjusted using the BH procedure. DailyMin, typical minutes of daily meditation; TtlYear, total number of years of meditation practice; Mful, trait mindfulness; PosAff, positive trait affect; NegAff, negative trait affect; Myst, mysticism; SA, self-actualization; Dvtn: devotion. ^*^p < 0.05; ^**^p < 0.01; ^***^p < 0.001.

### Multiple regression

Given the multiple significant correlations for devotion and the additional correlations among the variables, an exploratory multiple regression was performed to identify which relationships were most important for predicting devotion. Devotion was the outcome variable, and the other five psychological measures, two meditation variables and age (included as a covariate to control for age effects), were predictors. Only variables with sufficient independent variance would demonstrate significance in relation to devotion in a multiple regression. The multiple regression produced a significant model (*F*(8, 55) = 4.994, *p* < 0.001, *R*^2^ = 0.4207). Only one significant predictor, trait mindfulness (*t* = 3.147, *p* < 0.01), was present. Trait mindfulness accounted for the majority of the variance of this regression equation (34.3% of 42%). Self-actualization accounted for 3.7% which was not quite significant. The remainder was distributed among the other variables.

While there were multiple significant partial correlations among the predictor variables, the magnitudes were not sufficient to cause any substantial distortion of the regression equation through collinearity. The indicator that was used for collinearity was the variable inflation factor (VIF), with 5 as the indicator cutoff level. While some authors suggest that a VIF of 10 is a reasonable cutoff to eliminate confounding collinearity, a VIF of 2.5 can have sufficient collinearity to confound clearly identifying the contribution of correlated variables (Johnston et al., 2018). The highest VIF scores in this regression were less than 2.5: 2.17 for self-actualization and 2.16 for trait mindfulness. Since the VIFs were modest, multicollinearity is unlikely to explain the pattern of the results. Therefore, in the multiple regression, trait mindfulness was considered the only significant predictor of devotion. Because the design was cross-sectional, that association cannot be considered causal.

Exploring this further, when only trait mindfulness and self-actualization (the next strongest partial correlation with devotion) were used as predictors along with age, the model was more strongly significant (*F*(3, 60) = 13.42, *p* < 8.33e-07). Trait mindfulness remained the only significant predictor and was more strongly significant (*t* = 4.015, *p* < 6.42e-05), with a VIF = 1.63 for mindfulness and a VIF = 1.36 for self-actualization, age being even lower. Therefore, despite the significant partial correlation, self-actualization did not account for any relevant variance in these regressions with devotion.

## Discussion

It has been shown that trait mindfulness has a strong and primary relationship with devotion. While this exploratory analysis is unable to demonstrate causal relationships and even the observed findings require verification in future studies, subsequent sections will examine the broader relationship between devotion and mindfulness, and potential implications related to the significant regression. The nature of the additional expected outcomes for self-actualization, positive affect, negative affect, daily meditation and mystical experiences will also be discussed.

### Constant remembrance

The existence of common features between trait mindfulness and devotion is not as odd as one may initially think. Despite the clear conceptual differences between trait mindfulness and devotion, both include a similar process. Academically, mindfulness has been considered to include one-pointedness, or a unified mental focus (Mikulas, 2015). The traditional Buddhist conception of mindfulness provides a broader, but related framework. The Pali word “sati” used in early Buddhist texts has commonly been translated as “mindfulness.” Sati is the equivalent of the Sanskrit word “smrti,” which is commonly translated as “memory” (Sharf, 2014). While sati has developed greater connotations than just memory, the context of memory in relation to mindfulness still has relevance when expressed as “keeping in mind” (Anālayo, 2020, p. 241). The full expression of mindfulness has been described as unifying the mind and sustaining a mental flow to the level of one-pointedness, analogous to keeping something in mind without any distraction (Gunaratana, 1988; Huxter, 2015). While “one-pointedness” is not directly included within the CAMS-R trait mindfulness questions, the facets of attention, present focus and, to some extent, awareness, do provide measurements of varying levels of mental focus/distraction.

Similarly, at its fullest, devotion as expressed in AMTY has been described as “being merged in the constant thought of God” (Ánandamúrti, 1996, p. 35). This perspective expresses well what the DSES attempts to measure as “closeness to God.” The mental ideation underlying the “constant thought of God” has been referred to as “constant remembrance” or “dhruva smrti” in Sanskrit (Ánandamúrti, 1992a, p. 58) and as having unhindered, one-pointed ideation (Ánandamúrti, 2020). Elsewhere, Ánandamúrti referenced the principles of Buddha’s Noble Eightfold Path and equated dhruva smrti with the seventh principle, “samyak smrti,” in Sanskrit (or the Pali sammā sati, right mindfulness), which Ánandamúrti called “right memory” (Ánandamúrti, 1987, pp. 81–82). In that discourse, Ánandamúrti proceeded into a brief discussion of memory, not mindfulness. Despite Ánandamúrti’s disregard of the concept of mindfulness, he did proceed to emphasize, in a similar discourse on Buddha’s Eightfold Path at a different location more than a year later, that the “best smrti” is when one sustains an enduring mental focus on Parama Puruśa (Universal Consciousness/God), representing a peak devotional state (Ánandamúrti, 2011a, p. 273). Thus, both mindfulness and devotion emphasize the importance of being able to sustain a one-pointed focus. Those who have developed this attention capacity more fully would be expected to have higher scores for both trait mindfulness and devotion within this AMTY sample. One-pointedness within AMTY would likely include an affective component of devotional love that promoted focused attention. This may not be true in general within mindfulness practices, although a focus on compassion and loving kindness could generate a similar effect.

### Concentration and absorption

Concentration is closely related to both mindfulness and devotion. “Samādhi” is often used to refer to “concentration” in Buddhism, although in relation to “proper” practice, it would be “sammā samādhi” or “right concentration” (Kuan, 2008, p. 59). To be “established” in mindfulness, one must be “fully aware, concentrated, with one-pointed mind” (Kuan, 2008, p. 120). This one-pointedness in samādhi is necessary to achieve advanced states, called ‘jhānas’ (‘jhāna’ is the Pali version of the Sanskrit word ‘dhyāna’), described as ‘absorptions’ (Goleman, 1972; Anālayo, 2019a, 2019b). Thus, what has been called samādhi in Buddhist literature can be called a level of concentration necessary to achieve various deep experiential states of absorption that are at a different level than is typically accessible for novices or useful for basic mental health interventions.

Similarly, concentrating the mind in AMTY represents a steadying of the mind’s shifting focus to achieve one-pointedness (Ánandamúrti, 1988b). However, in AMTY, concentration is conceptualized as a precursor to what is called samādhi. Increased concentration is cultivated through multiple meditation practices within AMTY, especially AMTY versions of prāṇāyāma, dhāraṇā and dhyāna practices, the fourth, sixth and seventh limbs, of Patanjali’s eight-limb framework (Ánandamúrti, 1994b). Those practices lead to samādhi, the eighth and final limb. It is important to remember that the terminology (such as the various terms used to describe different types of samādhis) and practices (such as the versions of prāṇāyāma, dhāraṇā and dhyāna practices) used in AMTY are its own unique synthesis of various other systems, or its own unique innovation. Therefore, care has been taken to clarify AMTY terminology so that any bias of the participants in this study may be understood.

In AMTY, samādhi is also commonly translated by the word “absorption” (e.g., Ánandamúrti, 1999b). There are many different types of samādhis, just like there are many types of jhānas. Feuerstein (2008, p. 253) provides a useful comparison of two different Yoga terminologies for samādhi, one associated with Patanjali (a Samkhya system) and the other from Vedānta terminology that has been used in this case within AMTY. “In His Yogasūtra, Patanjali has elaborated a phenomenology of samādhi states that had been distilled from millennia of yogic experience. He distinguishes between two major species of samādhi, namely conscious ecstasy (samprajnāta-samādhi) and supraconscious ecstasy (asamprajnāta-samādhi). These correspond to the Vedānta distinction between formative ecstasy (savikalpa-samādhi) and formless ecstasy (nirvikalpa-samādhi) respectively”. Sarbacker (2005) considers notions of samādhi to have been developed in dialogue and discussion across Hindu and Buddhist disciplines, explaining the origin of their similarity. Further, Sarbacker uses the concept of “numinous” to more generally represent meditative absorptions that culminate in a subjective state of universal awareness. He uses the term “cessative” to similarly represent a state that goes beyond conscious phenomenal existence. “The connection of the numinous and experiential side of the spectrum with śamatha and the cessative and non-experiential side of the spectrum with nirvana, or nirodha, is a conception of samādhi that is paradigmatic to Classical Yoga as well as Buddhism” (Sarbacker, 2005, p. 130). He also considers these to be the “foundation upon which tantric theory was built” (Sarbacker, 2005, p. 135).

At the highest stage, the cessative principle in AMTY is called “nirvikalpa samādhi” which is considered to produce a “non-attributional” state, one lacking any qualities, freedom from all bondages, just pure existence, no subjective awareness and the end of suffering (Ánandamúrti, 1992b, 1993a, 1999a) and represents its own adaptation of terminology present in other Hindu oriented systems. Similarly, the elimination of suffering is a primary goal of traditional Buddhist meditation (Bodhi, 2011; Huxter, 2015). While Sarbacker (2005, p. 135) considers earlier Tantra to have emphasized the numinous dimension, he considers “late Indian and Tibetan contexts” of Tantra to have been “more focused on the cessative dimension”. The distinction between nirvikalpa samādhi versus savikalpa and other lesser samādhis (absorptions associated with various spiritual attainments) in yoga practices (including AMTY) has been considered relatively analogous to the distinction between a completely established vipassanā (insight) state, and samatha (serenity) and spiritual attainments associated jhāna absorptions (Sarbacker, 2005). This similarity in a culminating nondual and ineffable state for AMTY with at least some Buddhist traditions (Ánandamúrti, 1970; Thera, 2008; Kuan, 2020) demonstrates that both types of practices can generate higher levels that at their peak can similarly transcend subjective awareness. The impact of even partial mental stilling and related states of concentration, should increase the scores for both trait mindfulness and devotion in this study’s AMTY participants through facilitating mindful attention and present focus, and through facilitating stronger devotional focus. Thus, it is reasonable that a trait mindfulness measure and a measure of devotion would have a strong positive correlation and that trait mindfulness would predict devotion in a multiple regression for at least AMTY practitioners and potentially for individuals in other forms of Yoga practice.

### Additional influences

Other research has demonstrated increased well-being arising from both mindfulness training (Shapiro et al., 2008; Christie et al., 2017; Lomas et al., 2017, 2018) and spiritual meditation (Bormann et al., 2006; Ivtzan et al., 2013; Klein et al., 2016). In the present study, higher levels of both trait mindfulness and devotion were found to have significant partial correlations with decreased negative trait affect, increased positive trait affect, and greater self-actualization. Thus, the lifting of feelings and fulfillment of increasingly subtle personal needs suggest greater well-being, but those variables did not predict devotion in the regression. Well-being could alternatively be conceived as an outcome of both mindfulness and devotion. This hypothesis is consistent with research cited above. Further research with a large enough sample to include a path component in the analysis is needed to demonstrate this outcome within the AMTY and other populations.

Similarly, another potential influence of mindfulness and devotion may involve effort and motivation. Both mindfulness and devotion have been described as something that must be cultivated, requiring deliberate effort (Ánandamúrti, 1994c; Anālayo, 2019a, 2019b), although both authors indicated that at advanced stages, effort becomes unnecessary. While effort is difficult to quantify directly, the daily time spent meditating (DailyMin) could be a possible estimator of some aspects of effort. Since the partial correlation of DailyMin with devotion and mindfulness is comparatively weak and does not predict devotion in the regression, it may be more reasonable to think of elevated DailyMin as another potential outcome of elevated devotion and mindfulness. The subjective experience of greater devotion and mindfulness could produce greater inspiration to engage more strongly in the associated practices. For the vast majority of meditators, spending more time meditating each day would require greater motivation and effort. The weak correlations present suggest that this effect would be comparatively minor. This finding also needs to be confirmed in future research.

In contrast to expectations, there was no significant relationship between mystical experiences, represented by MS scores, and devotion or trait mindfulness. The participants in this study had a relatively high baseline MS score (*M* = 136.8, SD = 17.5). The AMTY MS scores are significantly different at high levels when compared to the findings (Keller et al., 2016) from a broad MS US sample (*n* = 1,113, *M* = 112.4, SD = 23.6, *t* = 8.14, *p* = 9.8e-16) and a broad German sample (*n* = 773, *M* = 114.2, SD = 31.8, *t* = 5.61, *p* = 2.7e-08). The maximum possible MS score is 160. The high AMTY baseline will limit variance and compromise the demonstration of any meaningful associations with other variables. Other issues could also have had influence on diminishing meaningful associations, such as the mean age (50.6 years old for AMTY versus 34.4 for an MS US sample and 43.2 years for the MS German sample). The timeframe referenced by the tests could be even more important. The CAMS-R measures a sustained mindfulness trait. Similarly, the DSES measures the persistence of one’s mental focus on one’s personal conception of the divine, analogous to a trait. In contrast, MS measures whether various typically infrequent mystical states have ever occurred or not. Since trait mindfulness and devotion are not related to isolated experiences, it makes sense that MS scores might not be related to them much, or even at all.

In contrast to DailyMin, TtlYear (total years of meditation) had no significant partial correlations. While some effort and motivation are certainly required to sustain a meditation practice over many years, the quality of that practice may have different characteristics and greater variability than that present for DailyMin. These negative findings require further research for verification.

### Limitations

The sample size in this study was relatively small and must be considered exploratory, requiring further research to confirm the findings. Future studies would benefit from a greater number of participants, which would allow for more complex statistical analysis, such as path analysis, which should include at least 100 participants. Designation of a proposed causal relationship at the onset of the research would also assist analysis.

The use of self-report questionnaires to measure trait mindfulness has been criticized for often having content vulnerable to misinterpretation and for measuring discrete psychological characteristics as if they provided a true definition and quantification of abstract conceptions (Grossman and Van Dam, 2011). Some of this criticism may be mitigated by the absence of completely novice controls in the present study. All participants in this study were practicing AMTY meditation, so any potential difference in interpreting test items should have been diminished.

The present study assessed status at only one time point, which limits what could be determined by this study. An alternative approach could be a longitudinal study, measuring the same group of individuals at two different times separated by a number of years and examining the degree of change in devotion, mindfulness and other variables. A longer time difference than typical meditation studies could diminish the expectation effects present in short studies.

Again, this is one small study and its findings require further substantiation.

## Conclusion

This study revealed a robust association between trait mindfulness and devotion among AMTY practitioners, with trait mindfulness being the unique predictor of devotion on a multiple regression. This finding indicates that cognitive attentional stability and affectively valenced devotional focus covary in experienced AMTY spiritual meditators. While cross-sectional data do not allow causal inference, these results are consistent with both mindfulness and devotion requiring the same underlying attentional processes, particularly a sustained, one-pointed focus. This type of focus is present in Buddhist descriptions of sammā sati (right mindfulness) and in AMTY’s concept of dhruva smrti (constant remembrance).

Trait mindfulness and devotion each had age-adjusted significant partial correlations with other variables commonly associated with well-being (higher positive affect, lower negative affect, greater self-actualization). These variables may represent a secondary outcome of greater well-being arising from elevated devotion and trait mindfulness. Daily meditation also has an age-adjusted significant partial correlation with both trait mindfulness and devotion, which may represent an additional secondary outcome related to enhanced effort/motivation.

Thus, there appear to be similarities in Buddhism-related mindfulness practices with the Yoga-related devotional practices of AMTY. The spiritual philosophies of both Buddhism and Yoga have been considered to be oriented toward achieving a closely related, if not identical, nondual and ineffable state of consciousness (Sarbacker, 2005). Despite the broad range of different religious/spiritual expressions, these similarities suggest the presence of a common underlying potential that may be available to all humans, whether in a religious/spiritual context, or in a secular context.

Generalizability is limited by using only an AMTY sample, by using self-report measures, and by the novel operationalization of devotion within this context. Future work should use longitudinal and multimethod designs, test measurement invariance across traditions, and evaluate a preregistered path.

## Data Availability

The datasets presented in this study can be found in online repositories. The names of the repository/repositories and accession number(s) can be found at: https://osf.io/24srp/.
